# Strategies for Enhancing Resilience in Horticultural Crops Against Combined Abiotic Stresses

**DOI:** 10.1111/ppl.70502

**Published:** 2025-09-07

**Authors:** Yaiza Padilla, Vasileios Fotopoulos, Georgia Ntatsi, Ángeles Calatayud, Consuelo Penella, Leo Sabatino, Maryam Mozafarian

**Affiliations:** ^1^ Departament de Biologia, Bioquimica I Ciències Naturals, Universitat JaumeI Castelló Castelló Spain; ^2^ Department of Agricultural Sciences Biotechnology and Food Science, Cyprus University of Technolo Limassol Cyprus; ^3^ Laboratory of Vegetable Production, Department of Crop Science Agricultural University of Athens Athens Greece; ^4^ Centro de Citricultura y Producción Vegetal Instituto Valenciano de Investigaciones Agrarias Moncada Valencia Spain; ^5^ Department of Agriculture, Food and Forestry Sciences University of Palermo Palermo Italy; ^6^ Department of Vegetable and Mushroom Growing Hungarian University of Agriculture and Life Sciences Budapest Hungary

**Keywords:** biostimulants, climate change, combined stress, grafting, nanotechnology, plant breeding

## Abstract

Horticultural crops are increasingly exposed to simultaneous abiotic stresses such as drought, salinity, and temperature extremes, which often exacerbate each other's effects, leading to severe yield and quality losses. Addressing these multifaceted challenges necessitates the development and application of integrated and innovative strategies. This review highlights recent advancements in methodologies to enhance the resilience of horticultural crops against combined abiotic stresses. Key approaches include breeding and selection of stress‐tolerant cultivars, grafting onto stress‐tolerant rootstocks, and priming strategies such as the application of nanoparticles and biostimulants, which have shown promise in modulating physiological and biochemical responses under stress conditions. These techniques collectively improve plant water status, enhance nutrient uptake efficiency, and upregulate antioxidant enzymatic activities, thereby mitigating oxidative damage and sustaining plant growth and productivity. By integrating these strategies, it is possible to optimize the physiological resilience and biochemical robustness of horticultural crops, ensuring stable yields and quality under increasingly challenging environmental conditions. These findings provide actionable insights into sustainable crop management and contribute to global efforts to enhance food security in the face of climate variability.

## 1 Introduction

Agricultural production is highly conditioned by the environmental fluctuations that affect plant growth and development. Abiotic stress, one of the most important adverse constraints that affect crop production and food security, accounts for more than 50% of agricultural losses (Oshunsanya et al. 2019). Among them, water scarcity, salinity, heavy metals, and extreme temperatures are constantly increasing under climate change conditions. Plants become affected at agronomic, physiological, and molecular levels, manifested mainly through a decrease in photosynthesis, an increase in oxidative processes, an alteration of membrane structures, enzymatic inactivation, hormonal and nutritional imbalances, leading to yield penalties when the plants' tolerance threshold is exceeded. These abiotic stresses are rarely found individually in real field conditions; instead, multiple stresses impact crops simultaneously, which may also interact with one another (Rillig et al. 2021). The interaction between these abiotic stresses could be synergistic, antagonistic, or additive. According to Zandalinas and Mittler (2022), (i) synergy occurs when the combined effects exceed that of the sum of the effects of each individual stressor; (ii) antagonistic interaction is when the overall impact of multiple stresses is less than the sum of the individual stresses; (iii) additive effects occur when the combined effect of multiple stresses is equal to that of their sum. The nature of these effects (synergistic, antagonistic, or additive) depends on the intensity of each individual stress involved in the combination, the order in which the stress is applied to the plant, and the plant species and development stage. The combined effects on the crops result in a specific/unique plant response that differs from individual stress responses. In fact, one of the biggest challenges for agriculture and scientific research is to develop new efficient strategies to improve the growth, development, and commercial production of crops under abiotic stresses in the context of climate change.

**FIGURE 1:**
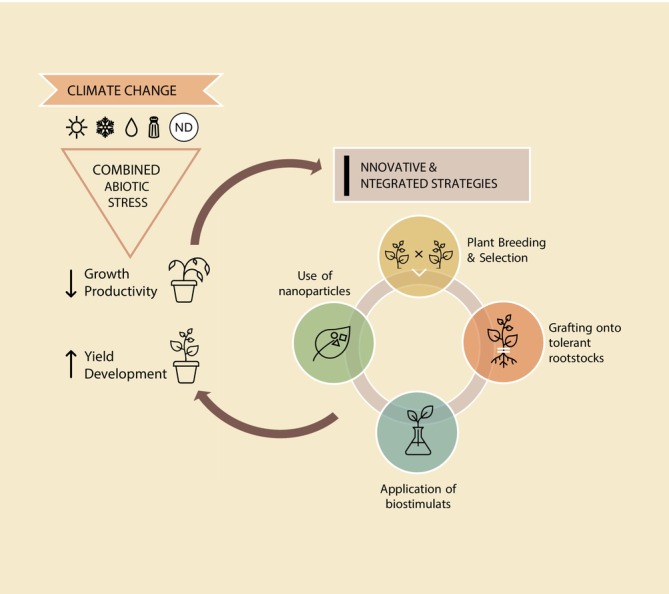
Strategies to enhance horticultural crop resilience under combined abiotic stress conditions. ND, nutrient deficiency.

In this regard, various approaches have been considered by the scientific community to overcome these negative effects of climate change. In this review, different methodologies and techniques that enhance tolerance to combined abiotic stresses in horticultural crops ranging from breeding, grafting technology to application of nanoparticles (NPs) and biostimulants are discussed (Figure 1).

**FIGURE 2:**
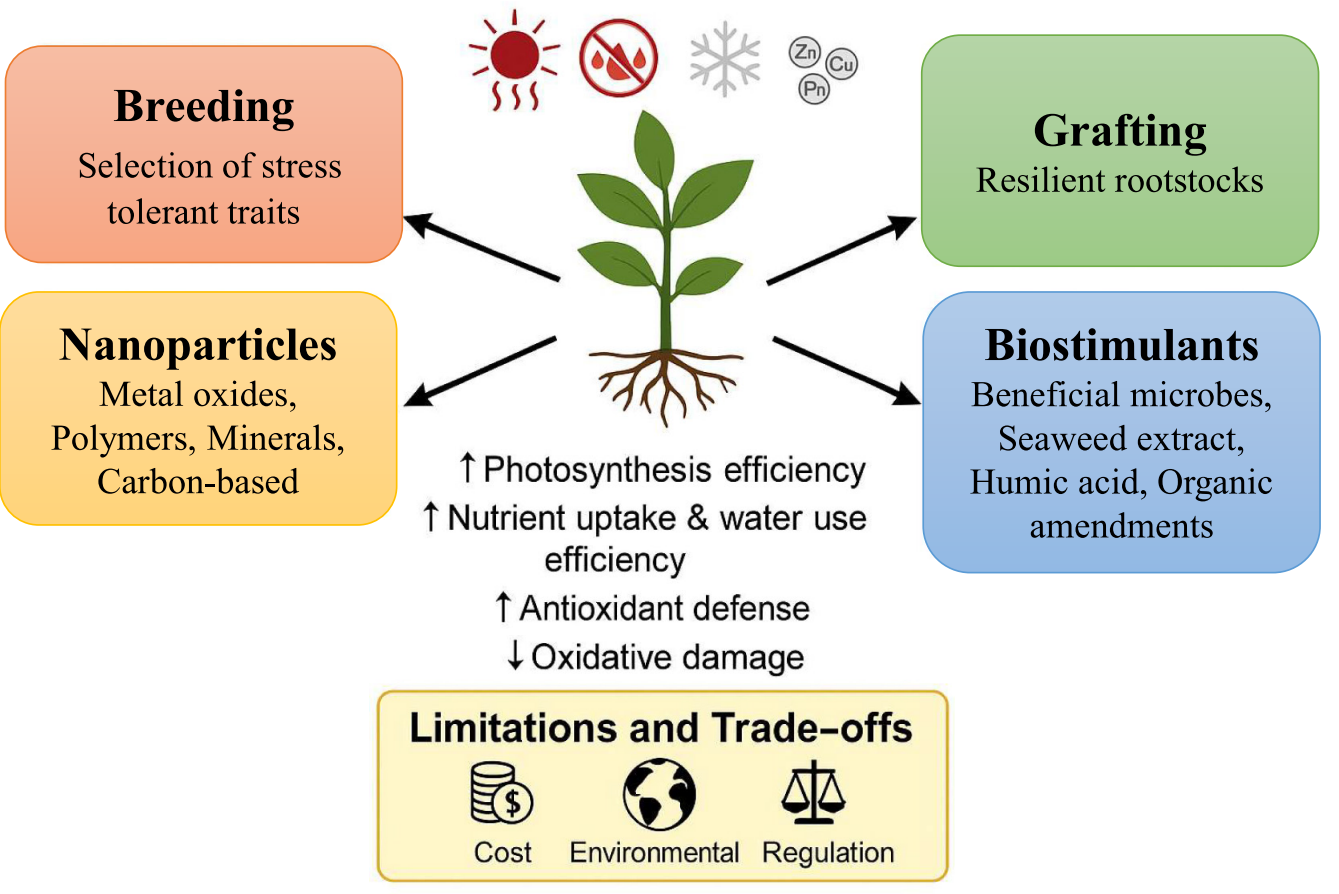
Integrated strategies to enhance resilience in horticultural crops under combined abiotic stresses, along with their key techniques and associated trade‐offs.

Strategies for enhancing resilience in horticultural crops against combined abiotic stresses is a complex challenge and surely the way to overcome it is to use a combination of all the tools described above. In this review, we analyze the use of these methods to reveal and/or mitigate the effect of abiotic stresses on horticultural crops, considering the complexity of the underlying physiological, biochemical, and molecular mechanisms (Figure 2). In the following sections, we will discuss up‐to‐date literature and the latest findings related to these approaches.

## 2 Breeding and Selection of Tolerant Cultivars

Under climatic stress conditions, it is crucial to develop “stress‐smart” crops that can withstand these conditions without considerable yield penalties (Joshi et al. 2024). Breeding and selecting tolerant cultivars are fundamental strategies to enhance resilience against these environmental constraints to meet the growing global food demand (Ghadirnezhad Shiade et al. 2023).

Breeding for abiotic stress tolerance is a multifaceted process that demands a thorough understanding of the physiological, biochemical, and molecular mechanisms governing plant stress responses (Gilliham et al. 2017). It also requires the strategic utilization of diverse genetic resources, like the exploitation of wild relatives and landraces as reservoirs of stress tolerance traits, which has also proven instrumental in expanding the genetic diversity of crop species (Brozynska et al. 2016).

### 2.1 Conventional Breeding Approaches

Traditional breeding methods have long been used to develop stress‐tolerant cultivars in horticultural crops. Techniques such as mass selection, recurrent selection, and hybridization remain essential despite their time‐consuming nature (Mishra et al. 1994). Genetic improvement in crops requires broad genetic variability, which serves as the foundation for breeding programs. This variability is found in different sources, including other commercial varieties, but it is especially abundant in traditional heirloom varieties and wild relatives (Gorbe et al. 2025; Dwivedi et al. 2019). These sources often exhibit key traits related to tolerance against various biotic and abiotic stresses, making them invaluable for crop improvement efforts. For example, crop wild relatives of carrot ( *Daucus carota* L.) have been selected for their tolerance to heat and drought simultaneous stresses (Simon et al. 2021).

For instance, breeding programs have successfully developed tomato ( *Solanum lycopersicum* L.) cultivars with simultaneous resistance to heat and drought stress, such as “Pusa Sadabahar” (Solankey et al. 2015). Similarly, lettuce ( *Lactuca sativa* L.) cultivars have been bred for tolerance to high temperatures and water deficit, ensuring stable yields under fluctuating environmental conditions (Hassan et al. 2021). Similarly, bell pepper ( *Capsicum annuum* L.) cultivars with enhanced salinity and drought tolerance have been successfully bred through extensive selection programs (Karim et al. 2021).

However, conventional breeding has limitations, including long breeding cycles, high costs, and pleiotropic effects, where a single gene influences multiple traits (Joshi et al. 2024). To accelerate the development of stress‐tolerant crops, integrating omics technologies, genome editing tools, and speed breeding is essential (Chakraborty et al. 2024; Raza et al. 2023).

### 2.2 Molecular Breeding and Omics‐Based Approaches

Conventional breeding approaches have been extensively employed to develop stress‐tolerant cultivars. However, the incorporation of advanced technologies has transformed the breeding landscape. These recent advancements in molecular breeding and omics technologies have significantly enhanced the capacity to develop stress‐resilient crops (Abdul Aziz and Masmoudi 2025). MAS and next‐generation sequencing (NGS) have facilitated the precise identification and incorporation of stress‐tolerant traits (Panahi et al. 2024), while transcriptomics, proteomics, and metabolomics have expanded the understanding of plant responses to combined abiotic stresses (Raza et al. 2024).

MAS has enabled the targeted selection of stress‐resilient traits through quantitative trait locus (QTL) mapping. This strategy has identified key genetic loci associated with drought and salinity tolerance in several crops, including tomato, cucumber, and lettuce (Mangal et al. 2024). In cucumber ( *Cucumis sativus* L.), MAS has led to the development of varieties with improved water use efficiency (WUE) (Younis et al. 2020), while in melon ( *Cucumis melo* L.), stress‐tolerant cultivars capable of maintaining yields under multiple environmental stressors, such as drought, high temperature, and salinity, have been selected (Chevilly et al. 2021; Shahwar et al. 2023; Oren et al. 2022).

Beyond MAS, genome‐wide association studies (GWAS) have provided new insights into the genetic basis of stress tolerance. In soybean ( *Glycine max* (L.) Merr.), integrating crop modeling with quantitative genetics has led to the identification of QTLs controlling oil yield plasticity under multiple abiotic stresses, including cold, drought, and nitrogen deficiency (Campbell and Stupar 2016). Recently, PvGT family genes in common bean ( *Phaseolus vulgaris* L.) have been involved; concretely, PvGT02 emerges as a potential candidate gene for breeding salt and drought tolerance (Raggi et al. 2024). A holistic genomic and functional analysis of bHLH genes and targeted promoter analysis in cucumber to respond to three abiotic stresses (NaCl, ABA and low‐temperature) revealed many cis‐elements responsive to multiple stresses and plant hormones (Joo et al. 2013). These approaches refine breeding strategies by elucidating stress tolerance mechanisms and identifying genes responsible for adaptive traits (González‐Guzmán et al. 2022).

The omics‐driven strategies offer critical insights into the intricate genetic architecture of stress tolerance traits, facilitating the identification of key genes and regulatory pathways involved in stress responses. Integrating transcriptomics, proteomics, and metabolomics has further expanded knowledge of plant responses to combined abiotic stresses. Transcriptomic studies have described the upregulation of drought‐responsive genes such as *DREB2A* (Dehydration‐Responsive Element‐Binding Protein 2A) in tomato, which plays a key role in dehydration responses (Xie et al. 2024), caused by simultaneous stresses such as heat and drought. Proteomic analyses demonstrated that YUCCA proteins are critical partners in auxin biosynthesis in plants; they are especially involved in cucumber under salinity and low temperature (Parvathi et al. 2022). Likewise, metabolomics studies in grapevine ( *Vitis vinifera* L.) have highlighted the accumulation of flavonoids and proline in response to combined drought and heat stress, underscoring the role of secondary metabolites in stress adaptation (Ferrandino et al. 2023).

### 2.3 Genome Editing

Genome editing provides a powerful approach to precisely modify stress‐related genes, offering an alternative to conventional breeding. CRISPR/Cas9 has emerged as the most widely used genome‐editing tool due to its efficiency and versatility (Zhu et al. 2023). It enables targeted modifications in genes that regulate multiple stress‐response mechanisms, allowing for the simultaneous improvement of drought, heat, and salinity tolerance (Jaganathan et al. 2018).

Genome editing has been successfully applied to engineer stress‐resilient crops by targeting genes with pleiotropic effects. For instance, the knockout of negative regulators *SlHyPRP1* and *SlDEA1* in tomato (*Solanum lycopersicum L*.) has been demonstrated to confer improved tolerance to both drought and salinity stresses (Saikia et al. 2024).

Beyond CRISPR/Cas9, novel genome‐editing technologies, such as base editing and prime editing, offer more precise modifications without introducing double‐strand breaks, reducing the likelihood of unintended mutations (Wang et al. 2022). Multiplex genome editing, which allows for the simultaneous targeting of multiple genes, represents a promising strategy for enhancing broad‐spectrum abiotic stress tolerance (Zhu et al. 2023).

Despite the great potential of genome editing, its successful application in horticultural crops to enhance resilience against combined abiotic stresses is still limited (Kumar et al. 2024). This is largely due to the relatively recent adoption of CRISPR/Cas9 and other genome‐editing technologies in plant breeding. However, as the technology continues to advance, along with improvements in delivery methods, regulatory frameworks, and multi‐gene targeting strategies, genome editing holds promising potential for the development of stress‐resilient horticultural crops in the near future. While regulatory changes are underway in Europe, the application of genome editing in horticultural crops remains restricted under the current GMO legislation, limiting its widespread adoption (Sundström et al. 2024).

## 3 Grafting as an Eco‐Technique to Overcome Abiotic Stresses

Grafting is an ancient, sustainable technique that joins together two plants: a scion, which constitutes the aerial part and accounts for fruit development; and a rootstock that provides the root system and could bring advantageous characteristics to the scion, such as tolerance to environmental stresses (Augstein and Melnyk 2025). This technique was originally applied to fruit trees and now it is almost essential in the citrus industry (Balfagón, Terán, et al. 2022). Regarding vegetable crops, the grafting technique was first employed in Japan and Korea to avoid soilborne diseases, which were aggravated in the 20th century due to the rise of continuous cropping and protected cultivation (Lee et al. 2010; Bie et al. 2017). Nowadays, grafting is a proven strategy to overcome adverse environmental conditions in horticultural crops and provide higher yields by the alleviation of stress‐related harm in the scion (Padilla et al. 2024; Ulas, Kılıç, and Abdullah 2025; Ulas, Kılıç, and Ulas 2025; Mozafarian et al. 2023; Mozafarian and Kappel 2020). The capacity of grafting to enhance tolerance in vegetable crops has been associated with various physiological and molecular improvements in grafted plants, including: (i) a more robust root system, (ii) increased efficiency in water and nutrient uptake, (iii) enhanced photosynthetic performance and water homeostasis, (iv) a reinforced antioxidative defense mechanism, (v) amplified hormonal signaling pathways, and (vi) extensive and long‐distance transport of mRNAs, small RNAs, and proteins (Albacete et al. 2015; Kumar et al. 2017).

### 3.1 Adaptation Strategies to Combined Abiotic Stresses in Roots

Grafting onto tolerant rootstocks brings several advantages to the scion such as better water and nutrient absorption, provided by an improved root system composed of bigger and/or deeper roots (Razi et al. 2024; Gisbert‐Mullor et al. 2023). However, there are relatively few studies of tolerance responses under combined abiotic stress focused on the root system, and even fewer studies involving grafted plants. It is crucial to study the adaptation strategies in roots under combined abiotic stress because it has been observed that plant response is specific and could differ from the accumulation of individual stress responses (Sánchez‐Bermúdez et al. 2022).

In chickpea, changes in root metabolome and transcriptome have been observed under combined N and P (nitrogen and phosphate) deficiencies (Nasr Esfahani et al. 2021, 2022) and these specific root metabolic profiles and gene expression changes do not correspond to the cumulative effects of N and P deficiency individually. The N and P deficiencies have also been studied in apple (Xie et al. 2023) and the authors find root architecture modifications in the combined deficiencies that are not comparable to the additive effects of the single deficiencies on root morphology As an adaptation to the N and P deficiency combination, grafted apples showed an increased root‐to‐shoot ratio, N and P partitioning in roots, and root length and volume. Tolerance to P deficiency in combination with water stress in peanut was manifested in increased water extraction related to higher root length, density, and biomass (Falalou et al. 2018), evidencing the major role of a robust root system in tolerance achievement. Similar tolerance responses were found in drought‐tolerant sugar beet under the combination of N deficiency and drought, which showed higher root biomass and increased content of root glycine betaine (Shaw et al. 2002), an osmoprotectant that also functions as an antioxidant compound (Ejaz et al. 2020).

Root antioxidant system activation is favored by tolerant rootstocks to compensate for the negative effects of reactive oxygen species (ROS) accumulation derived from cultivation in abiotic stress conditions (Padilla et al. 2021, 2023). In tomato, the combination of heat and salt stress resulted in increased roots ROS content counteracted by the stimulation of the antioxidant enzymes CAT and APX (catalase and ascorbate peroxidase) and the increase of antioxidant compounds such as GSH (reduced form of glutathione) and proline in roots (Sousa et al. 2022). An enhanced root antioxidant system in terms of CAT, SOD, and POD (superoxide dismutase and peroxidase) has also been described under the combination of cold and N and P deficiency in cucumber (Yan et al. 2013). Regarding cold and drought, Kaur et al. (2016) quantified more proline content and sustained membrane integrity in chickpea roots and described a positive effect when a pre‐treatment with drought was applied before cold stress. Other authors also observe an improved cold tolerance after a drought pre‐treatment via an antioxidant defense, manifested in a decreased ROS content in tomato roots (Ghanbari and Sayyari 2018).

Apart from the positive interaction mentioned on drought and cold stress, elevated CO_2_ has positive effects on roots in combination with drought, such as increased growth and respiration in pepper roots (del Amor et al. 2010), higher biomass and hydraulic conductivity in cucumber roots (Li et al. 2020). Similar beneficial effects on roots are found in the combination of high CO_2_ with salinity stress, as higher growth in peanut roots (Ratnakumar et al. 2013), lower Na^+^ content in citrus roots (García‐Sánchez and Syvertsen 2006), modulation of root water balance in broccoli by enhanced aquaporins activity (Zaghdoud et al. 2013) and improved root growth through hormonal regulation in tomato (Brito et al. 2020).

### 3.2 Rootstock Effect on Scion‐Related Traits

The adaptation mechanisms on roots from the tolerant rootstock under combined abiotic stresses could influence the aerial part of the plants towards tolerance, as reported by Zhang et al. (2016). A tight coordination must exist between rootstock and scion to regulate physiological and developmental responses to combined abiotic stress as a single plant (Sánchez‐Bermúdez et al. 2022).

Accordingly, several works have been conducted in citrus under heat and drought combined stress to identify tolerance‐related traits when using rootstocks. As an example, Jaskani et al. (2019) evaluated physiological responses in 10 different citrus rootstocks subjected to heat/drought stress combination and identified Brazilian sour orange as tolerant, given its higher root biomass and length complemented by maximum shoot biomass and height, resulting in a lower reduction in leaf chlorophylls and carotenoids. Similarly, Balfagón, Terán, et al. (2022) described tolerance to drought and heat stress when Carrizo was used as rootstock with beneficial effects on leaves of Cleopatra mandarin, observed in higher photosynthetic rate, less leaf damage and H_2_O_2_, and enhanced SOD and APX enzymatic activities. In succeeding studies, they also related this tolerance to increases in osmoprotectant sugars' content in leaves (raffinose and galactinol) and salicylic acid (SA) in leaves (Balfagón, Rambla, et al. 2022).

Concerning cold stress and low light combination, tolerance responses were noted when grafting cucumber onto tolerant rootstocks. Zhou et al. (2009) described an alleviation effect on photosynthetic rate using *Cucurbita ficifolia* rootstock, derived from an increased root sink strength accompanied by increased sucrose in cucumber leaves. In another experiment, Li et al. (2015) studied the adaptation strategies of three cucumber rootstocks and observed different tolerance degrees to cold and low light stress: cucumber grafted onto “Figleaf” had the highest tolerance in terms of less growth reduction, followed by the use of “Tielizhen” and “Kilameki”. Proline content and antioxidant enzyme activities (SOD, CAT and APX) followed the same pattern, being higher in Figleaf rootstock, together with lower electrolyte leakage and lipid peroxidation levels.

The tolerance of grafted plants under combined abiotic stress when tolerant rootstocks are used has been documented in several studies; however, there is a need to further determine the strategies involved in this tolerance with the aim of developing efficient tolerant rootstocks that overcome the combined stress harmful effects. Moreover, the lack of availability of rootstocks tolerant to combined abiotic stresses, the cost of two seeds per plant (the scion seed and the rootstock seed), as well as the labor cost of grafting per se, could hinder grafting adoption by farmers.

## 4 Application of Nanomaterials in Stress Management

Nanotechnology is an emerging and promising approach in modern agriculture, particularly for enhancing crop tolerance to abiotic stresses (Ioannou et al. 2020). It involves the manipulation of materials at the nanoscale (1–100 nm), where NPs—such as metal oxides, polymers, minerals, and carbon‐based materials—exhibit unique physicochemical characteristics that distinguish them from their bulk equivalents. These properties, including high surface area‐to‐volume ratio, increased chemical reactivity, and improved bioavailability, allow NPs to function as efficient carriers or active agents that can modulate plant physiological and biochemical responses under stress conditions (Srinivasan and Rana 2024; Fotopoulos and Gohari 2023). In addition, NPs can be synthesized with relatively low production costs, supporting their practical implementation at larger scales. Notably, their unique structural and functional attributes also make them highly suitable as smart nanocarriers (NCs) for the controlled delivery of bioactive compounds, including priming agents and agrochemicals (Gohari, Jiang, et al. 2024).

These NPs can be synthesized using physical, chemical, or biological methods. Among these, biological synthesis—also referred to as green synthesis—employs natural biological agents such as plant extracts, fungi, bacteria, and yeast. This method is widely recognized for being non‐toxic, environmentally sustainable, and relatively cost‐effective. Green synthesis strategies, in particular, offer plant‐based, fungal‐based, and microbial‐based routes to NP production, making them attractive for agricultural applications due to their biocompatibility and minimal environmental impact (Senthamizh et al. 2025).

Conventional approaches such as the use of chemical fertilizers, breeding for stress tolerance, and genetic modification have long been employed to mitigate abiotic stress in crops. However, these strategies often require long‐term implementation, are resource‐intensive, and may pose environmental challenges. In contrast, NPs represent an emerging and complementary tool, offering high bioavailability, controlled release properties, and the ability to activate plant defense mechanisms under stress conditions. Moreover, combining genome editing, speed breeding, and NPs enables the rapid development (within 1–2 years) of stress‐tolerant, high‐yielding elite crop varieties, offering an accelerated pathway toward food security and climate‐smart agriculture (Ahmar et al. 2021).

Priming is a promising strategy employed to enhance plant performance and resilience by enabling plants to better withstand environmental stresses. It involves a pre‐treatment process where plants are exposed to specific agents such as chemical molecules, beneficial microorganisms, and/or nanomaterials, which trigger a physiological and molecular “alert state” in the plant without causing actual stress damage (Conrath et al. 2015; Savvides et al. 2016). This heightened state of preparedness allows the plant to respond more rapidly and robustly when exposed to subsequent stressors, leading to improved tolerance to both biotic and abiotic stresses. Chemical priming, for example, can involve the application of phytohormones, ROS modulators, or other signaling compounds, while biological priming utilizes plant growth‐promoting rhizobacteria (PGPR) and fungi to induce systemic resistance. More recently, the use of nanomaterials in priming has emerged as a novel approach due to their ability to enhance the delivery and efficacy of active compounds, as well as directly influencing plant physiological responses (Savvides et al. 2016).

Priming strategies can be implemented at various stages of plant development and encompass a range of methods depending on crop species, production systems, economic feasibility, and specific stress conditions. Several delivery methods have been investigated for the application of NPs in priming and stress mitigation. These include foliar spraying, incubation of plant tissues with NPs, soil irrigation, and direct injection onto leaves (Yadav et al. 2024). Among these, seed priming—where seeds are treated with NPs before sowing—is widely used to enhance germination, seedling vigor, and stress tolerance. Foliar application is another efficient method in which NPs are directly applied to the leaf surface, facilitating rapid absorption and systemic action. Soil applications of NPs are also gaining attention due to their potential to improve nutrient availability, root development, and overall plant health by enabling root‐based uptake of the active compounds (Pradhan et al. 2025; Ding et al. 2023; Rhaman et al. 2022).

### 4.1 Role of NPs in Abiotic Stress Tolerance

NPs have demonstrated considerable potential in enhancing the tolerance of horticultural crops to a range of abiotic stresses, including drought, salinity, heat, and heavy metal toxicity (Table 1). Various NPs, including copper (Cu), silicon dioxide (SiO_2_), cerium oxide (CeO_2_), selenium (Se), iron oxide (Fe_3_O_4_ or Fe_2_O_3_), and zinc oxide (ZnO), have demonstrated considerable potential in promoting plant growth and improving tolerance to abiotic stresses (for examples, see Spanos et al. 2021; Table 1). For instance, ZnO NPs have been shown to improve drought resilience of cucumber and eggplant by enhancing antioxidant defenses, photosynthetic efficiency, and protecting cell membrane integrity (Ghani et al. 2022; Semida et al. 2021). Under salinity stress, Cu NPs in tomato and Se NPs in strawberry have been reported to maintain ionic balance, mitigate oxidative damage, and improve photosynthetic performance (Pérez‐Labrada et al. 2019; Zahedi et al. 2019). In response to heat stress, Si NPs improved membrane stability, water retention, and stimulated antioxidant activity in tomato (Khan et al. 2020). Similarly, the application of Se NPs has been found to alleviate heavy metal stress in tomato by limiting Cu accumulation, promoting nutrient uptake, and increasing overall biomass and fruit quality (Faizan et al. 2021). These findings highlight the promise of NPs as effective tools for sustainable horticulture by improving plant resilience and maintaining productivity under adverse environmental conditions.

**TABLE 1:** Example of NP types, stress targets, application methods, and physiological outcomes in horticultural crops.

Crop	Abiotic stress	Type of NPs	Application method	Key finding	References
Grapevine ( *Vitis vinifera* )	Salt	CeO_2_ NPs	Foliar	Improved growth, photosynthesis, antioxidant enzyme activity, and ion homeostasis; reduced oxidative stress.	Gohari et al. (2021)
Spearmint ( *Mentha spicata* )	Salt	Chitosan‐melatonin NPs	Foliar	Enhancing morphological traits, proline, antioxidant enzymatic activities, content of dominant constituents of EO profile	Gohari et al. (2022)
Corn salad ( *Valerianella locusta* )	Salt	Chitosan‐melatonin NPs	Seed priming	Enhancing morphophysiological traits, antioxidant enzymatic activities, polyphenolic compounds.	Gohari, Kulak, et al. (2024)
Sweet basil ( *Ocimum basilicum* )	Salt	Carbon NPs	Fertigation	Increased chlorophyll and carotenoid content and induced non‐enzymatic and enzymatic antioxidant components	Gohari et al. (2020)
Sweet basil ( *Ocimum basilicum* )	Salt	Octa‐aminopropyl polyhedral oligomeric silsesquioxanes NPs	Foliar	Increments in photosynthetic pigment, enhanced antioxidant system, and reduction in EL percentage, MDA and H_2_O_2_	Gohari et al. (2023)
Sweet orange (*Citrus* × *sinensis*)	Salt	Silicon NPs	Foliar	Improving root growth and photosynthesis, water status and regulating ion content	Mahmoud et al. (2022)
Pepper ( *Capsicum annuum* )	Heat	Proline NPs	Foliar	Improved growth traits, chlorophyll fluorescence, chlorophyll a, phenol, and proline	Masoumi, Haghighi, and Mozafarian (2024); Masoumi, Khosravi, et al. (2024)
Tomato ( *Solanum lycopersicum* )	Heat	Titanium oxide NPs	Foliar	Enhanced plant growth and photosynthesis efficiency	(Qi et al. 2013)
Hawthorns ( *Crataegus monogyna* )	Drought	Silicon NPs	Seed priming	Maintained rate of photosynthesis, stomatal conductance and enhanced biomass and water potential in xylem to deal with drought stress	Ashkavand et al. (2015)
Common bean ( *Phaseolus vulgaris* )	Salt	Silicon NPs	Seed priming	Enhanced vigor, percentage and speed of germination of seeds	Alsaeedi et al. (2017)
Okra ( *Abelmoschus esculentus* )	Salt	Zinc NPs	Foliar	Enhanced photosynthetic pigments, increased activities of SOD and CAT enzymes, and reduced accumulation of proline and total soluble sugars	Alabdallah and Alzahrani (2020)
Tomato ( *Solanum lycopersicum* )	Salt	Silicon NPs	Fertigation	Maintained concentration of chlorophylls, GSH, PAL activity, and vitamin C	Pinedo‐Guerrero et al. (2020)
Strawberry ( *Fragaria* × *ananassa* )	Salt	SA and iron NPs	Fertigation	Enhanced SOD and POD activity, increased protein and proline contents, and reduced H_2_O_2_ and MDA	Dedejani et al. (2021)
Tomato ( *Solanum lycopersicum* )	Salt	Silicon NPs	Seed priming	Improved germination percentage, germination rate and mean germination time	Haghighi et al. (2012)
Tomato ( *Solanum lycopersicum* )	Salt	Melatonin NPs	Foliar	Improved growth, water statues, antioxidant activities	Masoumi, Haghighi, and Mozafarian (2024); Masoumi, Khosravi, et al. (2024)
Tomato ( *Solanum lycopersicum* )	Drought	Silicon NPs	Seed priming	Improved germination rate	Haghighi et al. (2013)
Pepper ( *Capsicum annuum* )	Heat	5‐aminolevulinic acid NPs	Foliar	Improved growth, DPPH, proline, EL and antioxidant enzymes	Hallaji et al. (2024)
Lettuce ( *Lactuca sativa* )	Chilling	Chitosan–amino acid nanocomposites	Foliar	Increases in the total antioxidant activity and total phenolic content	Kalisz et al. (2024)
Bitter melon ( *Momordica charantia* )	Salt	Selenium‐chitosan NPs	Fertigation	Increased growth and photosynthesis parameters and expression of stress‐defense genes, increased antioxidant enzymatic activity	Sheikhalipour et al. (2021)
Moldavian balm ( *Dracocephalum moldavica* )	Salt	CeO_2_ NPs	Fertigation	Increased antioxidant enzyme activities, enhancement in EO content	Mohammadi et al. (2021)

However, additional research is needed to fully elucidate the role of NPs under combined stress conditions. Al‐Mayahi (2023) studied the combined effects of iron NPs and SA on the in vitro propagation of date palm under drought and salinity stress (PEG6000 + NaCl). The study found that applying 1 mg/L Fe NPs and 50 mg/L SA improved stress tolerance by enhancing antioxidant enzyme activity (SOD, APX) and promoting solute accumulation. This suggests their protective role in reducing oxidative stress and improving plant resilience. Malekzadeh et al. (2023) demonstrated that graphene oxide NPs alleviate salt and alkalinity stress in strawberry plants by improving gas exchange and photosynthetic efficiency. This enhancement in photosynthetic performance contributed to better overall plant resilience.

The synergistic potential of combining NPs with microorganisms to promote abiotic stress tolerance also constitutes an interesting research direction. Recent studies have highlighted the potential of combining NPs with other agronomic or biological tools to enhance plant resilience under abiotic stress. In particular, NPs have been shown to act synergistically with plant growth regulators, signaling molecules, and beneficial microbes to amplify stress tolerance responses. Khan et al. (2024) reviewed the interactive effects of NPs in conjunction with phytohormones, PGPR, and fungi, emphasizing their collective role in promoting growth and mitigating abiotic stresses. This synergy improves the activity of antioxidant enzymes, increases the availability and uptake of mineral nutrients, and strengthens plant stress tolerance responses. As a result, plants exhibit reduced oxidative damage, enhanced physiological performance, and greater overall vigor, ultimately contributing to improved agricultural productivity. Sayed et al. (2024) investigated the combined application of *Arbuscular mycorrhiza* fungi (AMF) with foliar application of Zn NPs and Se‐NPs in chili pepper growth under cold stress. The application of ZnO‐NPs + Se‐NPs combined with AMF enhanced transpiration rate, chlorophyll content, stomatal conductance, photosynthetic efficiency, vegetative growth, flowering, productivity, mineral content, antioxidant enzymes, and nitrogen metabolism. Moreover, it reduced stress indicators such as abscisic acid (ABA), malondialdehyde (MDA), and hydrogen peroxide (H_2_O_2_), making it the most effective treatment across both growing seasons. Similarly, Ostadi et al. (2022) demonstrated that the co‐application of AMF and titanium dioxide (TiO_2_) NPs in sage ( *Salvia officinalis* ) under drought conditions resulted in improved dry matter yield, nutrient uptake, WUE, and essential oil (EO) production. These findings underscore the potential of integrating nanotechnology with microbial inoculants and other biological interventions to enhance stress resilience in horticultural crops.

### 4.2 Mechanisms of NPs Uptake and Metabolism

The uptake and translocation of NPs in plants are influenced by several physicochemical factors, including particle size, surface charge, shape, and surface functionalization or coating. Following foliar application, NPs typically enter plant tissues through stomatal openings or by diffusing across the cuticle. In contrast, root‐absorbed NPs are taken up through the apoplastic or symplastic transport routes and can subsequently enter the xylem for systemic distribution throughout the plant (Khan et al. 2024). After entry into plant tissues, NPs may accumulate in organelles such as chloroplasts, where they contribute to enhanced photosynthetic efficiency and enzymatic activation. For example, ZnO NPs have been observed to localize in chloroplasts, resulting in increased photosynthetic capacity and upregulation of stress‐responsive enzymes (Zahedi et al. 2019). Nevertheless, the mechanisms governing NP mobility, organ‐specific distribution, and bioavailability under field conditions remain insufficiently understood and warrant further investigation.

In addition to their physical transport, NPs influence various metabolic and genetic processes that contribute to improved stress tolerance. These include the modulation of gene expression, activation of signaling cascades, and stimulation of the plant's antioxidant defense system. Notably, NPs have been shown to enhance the activity of key antioxidant enzymes such as SOD, CAT, and APX, which play critical roles in scavenging ROS and reducing oxidative stress (Yadav et al. 2024). Furthermore, they facilitate improved nutrient uptake and metabolic regulation, collectively supporting plant performance under adverse environmental conditions (Ping et al. 2024).

## 5 Role of Biostimulants in Enhancing Crop Resilience Against Combined Abiotic Stresses

Biostimulants are natural or synthetic substances that enhance plant growth, crop production, and resilience to single abiotic stresses such as drought, salinity, and extreme temperatures (Ntanasi et al. 2024; Amerian et al. 2024; Sabatino et al. 2023; Khani et al. 2025). Indeed, biostimulant products containing bioactive molecules with external application on the plants have a beneficial effect and improve their capability to face adverse environmental conditions, acting on primary or secondary metabolism (Bulgari et al. 2019). However, only a limited number of research reports are available in the literature demonstrating the role of biostimulants in the mitigation of combined stresses (Begum et al. 2019). Biostimulants can be categorized into several types, including protein hydrolysates (PHs), seaweed extract (SW), humic substances, microbial inoculants (e.g., AMF and PGPR), and plant‐derived compounds like melatonin and γ‐aminobutyric acid (GABA) (Vougeleka et al. 2024; Savvas et al. 2024; Vultaggio et al. 2024; Campana et al. 2025). Arbuscular mycorrhizal fungi (AMF) are symbiotic fungi that colonize plant roots and improve water and nutrient uptake while modulating plant hormonal and antioxidant responses under stress. Plant growth‐promoting rhizobacteria (PGPR) are beneficial bacteria inhabiting the rhizosphere that enhance plant growth through mechanisms such as phytohormone production and induction of systemic tolerance (Begum et al. 2019; Rouphael and Colla 2020).

Recent studies using ‘‐omics’ approaches (e.g., transcriptomics, proteomics, metabolomics) have begun to unravel the complex molecular interactions between biostimulants and plant stress responses, revealing extensive alterations in gene expression patterns and metabolic networks that collectively enhance resilience against multiple stresses (Bhupenchandra et al. 2022; Xu et al. 2020). These biostimulatory effects involve nutrient assimilation and transport, modulation of phyto‐hormonal profiles, photosynthesis and osmolyte accumulation, antioxidant activity and phenolic compounds enhancement (Table 2). These mechanisms are often interconnected and contribute to overall stress mitigation.

**TABLE 2:** Research on biostimulants in different crops and their effect on tolerance to combined abiotic stresses.

Type of biostimulant	Crop	Abiotic stress	Beneficial effect	References
Microbial inoculant				
AMF (*Septoglomus constrictum*)	Tomato	Water deficit and heat stress	Improved photosynthetic performance, leaf water potential and RWC. Higher nutrient uptake and biomass.	Duc et al. (2018)
PGPR ( *Azotobacter chroococcum* 76A)	Tomato	Salinity and sub‐optimal nutrition	Contribution to nitrogen metabolism and nutrient uptake	Van Oosten et al. (2018)
PGPR (with grafting)	Tomato	Water and nutrient stress	Improved nitrogen acquisition, increased root surface area and stimulation of nutrient mobilization.	Kalozoumis, Savvas, et al. (2021)
Seaweed extracts and protein hydrolysates				
Algae and glycine betaine‐based	Olive trees	Water deficit and high temperature	Improved RWC, better stomatal conductance and biomass production. Higher nutrient availability and utilization	Graziani et al. (2022)
Seaweed extract	Wild rocket	Nitrogen and water stress	Improved WUE, NUE, PE, PFPn and nitrogen AE, optimizing nitrogen utilization	Candido et al. (2023)
SE and protein hydrolysate	Sweet basil	Nitrogen deficiency and Water deficit stress	Increased NUE, WP, polyphenols and volatile compounds	Consentino et al. (2023)
SE	Wild rocket	Water and nitrogen deficit	Higher chlorophyll content, net assimilation and antioxidant enzymes	Schiattone et al. (2023)
PH	Tomato	Water deficit and heat stress	Increased net photosynthetic rate, Fv/fm, antioxidant content and lower H_2_O_2_	Francesca et al. (2022)
PH	Soybean, chickpea, and chili	Water deficit and high air temperature	Increased proline content, RWC, PSII efficiency and chlorophyll content leading to improved yield	Mamatha et al. (2023)
PH	*Cichorium spinosum* L.	Nitrogen deficiency and drought	Enhanced leaf number, leaf area, and leaf fresh weight, but ineffective in alleviating drought stress.	Voutsinos‐Frantzis et al. (2024)
Organic amendments, plant extracts and plant‐derived compounds				
Acidified leguminous compost and silymarin extract	*Atriplex nummularia*	Saline‐calcareous stress	Enhanced antioxidant system, improved nutrient uptake (N, P, K), enhanced photosynthetic rate, maintaining photosynthetic efficiency and nutrient assimilation.	Rady et al. (2023)
Melatonin	Tomato	Salinity and heat stress	Upregulated expression of antioxidant‐related genes (APX, GR, GPX and Ph‐GPX), improved photosynthetic parameters and enhanced PSII function.	Martinez et al. (2018)
GABA	Sunflower	Water deficit and heat stress	Enhanced activities of SOD, POD, GR, and APX. Reduction in oxidative stress markers, enhanced soluble sugars, increased chlorophyll and improved photosynthetic attributes.	Abdel Razik et al. (2021)
Humic acid	Mungbean	Water deficit and salinity stress	Upregulation of drought‐tolerant and salinity‐related genes. Increased chlorophyll, photosynthesis rate, stomatal conductance and transpiration rate.	Alsamadany (2022)
Strigolactone‐based	Tomato	Nutrient and water stress	Maintain nitrate levels in the root zone, improved nutrient retention and utilization	Kalozoumis, Vourdas, et al. (2021)
Non‐hormonal biostimulant (antioxidant and amino acid‐based)	Cowpea	Savanna conditions (nitrogen limitation and drought)	Contributes organic nitrogen, sustaining plant growth and biomass production.	Atta‐Boateng and Berlyn (2021)

### 5.1 Microbial Inoculants (AMF and PGPR)

Biostimulants significantly enhance nutrient acquisition and utilization, particularly under combined stress conditions. A key mechanism by which biostimulants enhance nutrient‐related tolerance to combined stresses is through microbial symbiosis. AMF, such as *Septoglomus constrictum*, has been found to improve nutrient uptake and biomass production in tomato plants subjected to drought and heat stress (Duc et al. 2018). The symbiotic relationship between the fungi and the host plant resulted in improved photosynthetic performance by enhancing chlorophyll content, stomatal conductance, and PSII (photosystem II) efficiency. The inoculation with *S. constrictum* also enhanced tomato leaf water potential and RWC.

Van Oosten et al. (2018) found that *Azotobacter chroococcum* 76A improves nitrogen metabolism and nutrient uptake in tomato plants under salinity stress, particularly when combined with suboptimal nutritional conditions. Microbial biostimulants, such as fungi and PGPR, modulate hormonal signaling to enhance stress tolerance. These hormonal adjustments improve root development and nutrient assimilation, contributing to enhanced stress resilience. According to Kalozoumis, Savvas, et al. (2021), PGPR and grafting improve nitrogen acquisition in tomato plants under combined water and nutrient stress. The authors observed that PGPR treatments maintained leaf nitrogen concentrations at levels similar to nonstressed plants, likely due to increased root surface area and stimulation of metabolic processes involved in nutrient mobilization.

### 5.2 Seaweed Extracts and Protein Hydrolysates

The application of glycine betaine and algae‐based biostimulants improves RWC in olive trees under drought and high temperature stress (Graziani et al. 2022). This improvement in water relations is related to better stomatal conductance and biomass production. These biostimulants also improve nutrient availability and utilization, enhancing plant resilience. Candido et al. (2023) demonstrated that SW improved WUE, nitrogen use efficiency (NUE), physiological efficiency (PE) by 20%, partial factor productivity (PFPn) and nitrogen agronomic efficiency (AE) in wild rocket subjected to nitrogen and water stress, highlighting their role in optimizing nitrogen utilization under stress conditions. Furthermore, Consentino et al. (2023) reported that SW and PH increased NUE, water productivity (WP), polyphenols, and volatile compounds in sweet basil under nitrogen deficiency and drought stress.

Photosynthesis is often severely impaired by abiotic stress, leading to reduced plant growth and productivity. SWs improve chlorophyll content, net assimilation, and antioxidant enzymes in wild rocket under water and nitrogen deficit (Schiattone et al. 2023). Additionally, PH‐based biostimulants increase the net photosynthetic rate, maximal efficiency of PSII photochemistry (^Fv^/_fm_), and the antioxidant content while they reduce H_2_O_2_ accumulation in tomato plants under drought and heat stress (Francesca et al. 2022). This was attributed to the presence of glycine betaine in the biostimulants, which stabilizes the photosynthetic machinery. Biostimulants promote the accumulation of osmolytes, such as glycine betaine, and soluble sugars, which help stabilize cellular structures and maintain osmotic balance under stress. PHs, for instance, increase proline content, RWC, PSII efficiency, and chlorophyll content in soybean, chickpea, and chili plants exposed to water deficit and high air temperature, leading to improved yield (Mamatha et al. 2023).

Moreover, biostimulants contain bioactive compounds that stimulate the biosynthesis of endogenous phytohormones, promoting plant growth and stress adaptation. Rouphael et al. (2017) suggested that biostimulants with free amino acids or peptides can enhance phyto‐hormonal biosynthesis, while others, according to Matsuo et al. (2012), exhibit cytokinin‐like activity, boosting cell proliferation and growth. A protein hydrolysate biostimulant can partially mitigate the effects of N deficiency by enhancing leaf number, leaf area, and leaf fresh weight in *Cichorium spinosum* L. plants, as reported by Voutsinos‐Frantzis et al. (2024) although it is ineffective in alleviating drought stress.

### 5.3 Organic Amendments, Plant Extracts and Plant‐Derived Compounds

The use of a silymarin‐enriched biostimulant combined with acidified leguminous compost was reported to enhance the antioxidant system of *Atriplex nummularia* plants subjected to saline‐calcareous stress (Rady et al. 2023). These biostimulants significantly improve nutrient uptake (N, P, K) and enhance the photosynthetic rate, demonstrating their role in maintaining photosynthetic efficiency and nutrient assimilation.

Martinez et al. (2018) found that melatonin treatment upregulates the expression of antioxidant‐related genes, such as APX, glutathione reductase (GR), glutathione peroxidase (GPX), and phospholipid hydroperoxide glutathione peroxidase (Ph‐GPX), in tomato plants subjected to combined salinity and heat stress. Moreover, the authors found that melatonin treatment improves photosynthesis parameters, including stomatal conductance and electron transport rate, and enhances PSII function, contributing to better photosynthetic efficiency. Abdel Razik et al. (2021) also observed that GABA treatment enhances the activities of antioxidant enzymes, including SOD, POD, GR, and APX, in sunflower plants under drought and heat stress. This was accompanied by a reduction in oxidative stress markers, such as H_2_O_2_ and MDA, indicating effective ROS scavenging. The authors also observed enhanced soluble sugars, increased chlorophyll content, and improved photosynthetic attributes, such as photosynthetic rate, stomatal conductance, and quantum efficiency.

In addition, plants activate genetic mechanisms, such as the upregulation of drought‐tolerant (*VrDREB2A*, *VrbZIP17*, *VrHsfA6a*) and salinity‐related (*VrWRKY73*, *VrUBC1*, *VrNHX1*) genes, to cope with abiotic stresses by accumulating osmolytes and maintaining osmotic balance. The application of humic acid modulates these genetic responses and increases total chlorophyll, photosynthesis rate, stomatal conductance, and transpiration rate under combined drought and salinity stress, enhancing stress resilience and productivity in the mungbean genotype PRI‐Mung‐2018 (Alsamadany 2022).

Furthermore, there is evidence that a strigolactone‐based biostimulant maintains nitrate levels in the root zone of tomato plants exposed to nutrient and water stress, suggesting improved nutrient retention and utilization as another key mechanism of biostimulant application under combined stress (Kalozoumis, Vourdas, et al. 2021). Atta‐Boateng and Berlyn (2021) also found that a non‐hormonal biostimulant (antioxidant and amino acid‐based) contributes to organic nitrogen, alleviating nitrogen limitation in cowpea under savanna conditions, thereby sustaining plant growth and biomass production.

## 6 Potential Limitations, Challenges

Despite the promise of nanotechnology, biostimulants, grafting, and breeding in enhancing crop resilience, several limitations remain. Nanoparticles improve stress tolerance but raise concerns about long‐term soil accumulation, microbial disruption, and food chain entry, while production and application costs may limit large‐scale use (Tripathi et al. 2017). Biostimulants show variable efficacy depending on crop genotype and environment, and the lack of standardized formulations reduces reproducibility (Rouphael and Colla 2020; Bulgari et al. 2019). Grafting is effective but labor‐intensive and costly, requiring compatible rootstock–scion combinations (Lee et al. 2010; Bie et al. 2017). Breeding and genome editing offer durable solutions but face long development times and regulatory restrictions (Sundström et al. 2024).

Overall, these strategies must be evaluated not only for biological efficacy but also for economic feasibility and environmental safety, ensuring their practical implementation in diverse horticultural systems.

## 7 Conclusions and Future Perspectives

Climate change‐induced abiotic stresses—such as drought, salinity, heat, and nutrient imbalances—pose a significant threat to global horticultural productivity. These stresses often occur simultaneously in the field, creating complex plant responses that cannot be addressed by single‐stress strategies. This review has explored different approaches, including advanced breeding techniques, grafting technologies, application of NPs, biostimulants, and PGRs, all of which contribute to strengthening plant resilience against multifactorial abiotic stress conditions.

Looking ahead, future research should focus on decoding the specific physiological, biochemical, and molecular mechanisms that underlie plant responses to multiple concurrent stresses. These responses often differ fundamentally from those triggered by individual stressors, emphasizing the need for complex, tailored solutions. Developing new phenotyping tools and screening platforms that reflect field‐like stress combinations will accelerate the identification of resilient genotypes. At the same time, optimizing the dosage, timing, and integration of inputs such as biostimulants, NPs, and PGRs could unlock synergistic effects that enhance overall stress mitigation. Cross‐strategy combinations—such as using NPs with beneficial microbes or pairing biostimulants with resilient rootstocks—represent particularly promising areas for exploration under combined stresses. Further research is needed for optimized formulation and application of these strategies under realistic multifactorial scenarios to achieve optimal cost–benefit for growers.

## Author Contributions

**Yaiza Padilla:** writing – review and editing, writing – original draft, visualization, and conceptualization. **Vasileios Fotopoulos:** writing – review and editing, writing – original draft, visualization, and conceptualization. **Georgia Ntatsi:** writing – review and editing, writing – original draft, visualization, and conceptualization. **Ángeles Calatayud:** writing – review and editing, writing – original draft, visualization, and conceptualization. **Consuelo Penella:** writing – review and editing, writing – original draft, visualization, and conceptualization. **Leo Sabatino:** writing – review and editing, writing – original draft, visualization, and conceptualization. **Maryam Mozafarian:** writing – review and editing, writing – original draft, visualization, and conceptualization.

## Data Availability

The authors have nothing to report.
